# Trastuzumab and first-line taxane chemotherapy in metastatic breast cancer patients with a HER2-negative tumor and HER2-positive circulating tumor cells: a phase II trial

**DOI:** 10.1007/s10549-023-07231-4

**Published:** 2024-01-31

**Authors:** Noortje Verschoor, Manouk K. Bos, Ingeborg E. de Kruijff, Mai N. Van, Jaco Kraan, Jan C. Drooger, Johanna M. Zuetenhorst, Saskia M. Wilting, Stefan Sleijfer, Agnes Jager, John W. M. Martens

**Affiliations:** 1 Department of Medical Oncology, Erasmus MC Cancer Institute, Rotterdam, The Netherlands; 2 Department of Internal Medicine, Breast Cancer Center South Holland South, Ikazia Hospital, Rotterdam, The Netherlands; 3 Department of Medical Oncology, Franciscus Gasthuis & Vlietland, Rotterdam/Schiedam, The Netherlands

**Keywords:** Circulating tumor cells, HER2, Trastuzumab, Metastatic breast cancer, Predictive biomarker

## Abstract

**Purpose:**

HER2 overexpressing circulating tumor cells (CTCs) are observed in up to 25% of HER2-negative metastatic breast cancer patients. Since targeted anti-HER2 therapy has drastically improved clinical outcomes of patients with HER2-positive breast cancer, we hypothesized that patients with HER2 overexpressing CTCs might benefit from the addition of trastuzumab to chemotherapy.

**Methods:**

In this single-arm, phase II trial, patients with HER2-positive CTCs received trastuzumab as addition to first-line treatment with taxane chemotherapy. Patients with detectable CTCs but without HER2 overexpression that received taxane chemotherapy only, were used as control group. The primary outcome measure was progression-free rate at 6 months (PFR6), with a target of 80%. In November 2022, the study was terminated early due to slow patient accrual.

**Results:**

63 patients were screened, of which eight patients had HER2-positive CTCs and were treated with trastuzumab. The median number of CTCs was 15 per 7.5 ml of blood (range 1–131) in patients with HER2-positive CTCs, compared to median 5 (range 1–1047) in the control group. PFR6 was 50% in the trastuzumab group and 54% in the taxane monotherapy group, with no significant difference in median PFS (8 versus 9 months, p = 0.51).

**Conclusion:**

No clinical benefit of trastuzumab was observed, although this study was performed in a limited number of patients. Additionally, we observed a strong correlation between the number of evaluable CTCs and the presence of HER2-positive CTCs. We argue that randomized studies investigating agents that are proven to be solely effective in the HER2-positive patient group in patients with HER2-positive CTCs and HER2-negative tissue are currently infeasible. Several factors contribute to this impracticality, including the need for more stringent thresholds, and the rapidly evolving landscape of cancer treatments.

**Supplementary Information:**

The online version contains supplementary material available at 10.1007/s10549-023-07231-4.

## Introduction

With the introduction of anti-HER2 targeted therapy, the clinical outcome of patients with HER2-positive breast cancer has greatly improved. HER2-positive disease is defined by receptor overexpression, caused by amplification of the *ERBB2* gene. According to current guidelines, HER2-status is determined by semi-quantitative determination of overexpression by immunohistochemistry, complemented by in situ hybridization if necessary[1]. HER2-positivity is an inherently aggressive trait and is present in 10–15% of all primary invasive breast cancers. Heterogeneity between lesions has been described when the disease advances to the metastatic state, with conversion from HER2-negative to HER2-positive state in up to 10%[2–4]. Retrospective research suggests that patients with discordant HER2-receptor status between the primary and metastatic lesion derive benefit from HER2-targeting treatment, although this has to be confirmed in larger series[5]. The current guidelines for metastatic breast cancer recommend tissue re-evaluation prior to initiating a new line of systemic treatment if possible, and to treat patients with targeted therapy if HER2-is positive in at least one biopsy[6]. It is however not always feasible to biopsy metastatic lesions to re-evaluate HER2-status. Non-invasive evaluation of the HER2-status on circulating tumor cells (CTCs) is proposed as a possible solution. The general hypothesis is that CTCs detach from all tumor lesions and therefore provide a better reflection of receptor status of all metastatic lesions[7]. However, CTCs are rare events, which are detected against a vast background of lymphocytes, which complicates evaluation of genomic markers at a single cell level. Riethdorf et al. therefore proposed an immunostaining method to evaluate HER2-expression on CTCs, that can be combined with the FDA-approved CellSearch method, the current gold standard to retrieve CTCs, due to its proven clinical validity[8, 9]. Interestingly, in cohort studies applying this immunostaining method, HER2-overexpressing CTCs were observed in up to 25% of patients with metastatic breast cancer (MBC) who had a HER2-negative primary tumor, thereby exceeding the reported 10% in tissue[10]. It was previously shown in a small retrospective set with that HER2-negative patients with HER2-overexpressing CTCs might benefit from anti-HER2 therapy in the form of the monoclonal antibody trastuzumab[11]. The underlying rationale is that these overexpressing CTCs reflect heterogeneity in receptor status of all metastatic lesions. The primary objective of this phase II trial was therefore to prospectively determine if MBC patients with a HER2-negative primary tumor but HER2-positive CTCs benefit from trastuzumab-containing taxane chemotherapy.

## Methods

### Patients and treatments

Patients with metastatic breast cancer that had a HER2-negative tumor, irrespective of ER-status, and that were starting first-line taxane-based chemotherapy, were asked to participate. HER2-negative was defined as immunohistochemistry 0, 1 + or 2 + on all available tumor tissue, primary and/or metastatic. In case of 2 + , FISH was performed of which the HER2-ratio should be < 2.0, according to ASCO/CAP guidelines[1]. Other inclusion criteria were ECOG performance status of 0–2, and cardiac left-ventricular ejection (LVEF) ≥ 45%. Patients with ER-positive (ER +) disease could have received any number of endocrine treatment regimens before the start of taxanes. Exclusion criteria were: adjuvant chemotherapy within 6 months before start of the study, endocrine therapy up to 1 week before start of taxanes, and symptomatic CNS metastases.

Before the start of treatment, 7.5 ml peripheral blood was collected in CellSave preservation tubes and CTC enumeration was performed within 96 h by the CellSearch system, with addition of an immunofluorescent anti-HER2 antibody according to previous reporting by Riethdorf et al.[9]. Patients were eligible for the treatment phase of the study if at least one HER2-positive CTC was found, defined as semi-quantitative staining 2 + or 3 + . This was scored by two independent, trained observers. In the treatment phase, trastuzumab was added according to standard of care to either docetaxel or paclitaxel, at the physicians choice. Patients received up to six cycles of treatment with taxane therapy. After this, treatment with trastuzumab and if applicable endocrine treatment, was continued until disease progression. Patients were evaluated after six months according to RECIST 1.1 or in case of poorly evaluable disease with other suitable techniques (e.g., bone scan or serum biomarkers), unless disease progression was observed before this time point. All patients provided written informed consent before participating in this study.

### Course of the trial

The inclusion started in March of 2015. However, over the last decades, capecitabine is used more often as a first line therapy, due to its favorable toxicity profile. We did not amend for this, because the combination of capecitabine + trastuzumab is inferior according to guidelines[6]. As a result, inclusion dropped to such a rate that the trial would have to run for dozens of years. Therefore, the trial was closed for inclusion in October 2022. At this time 8 patients were included in the treatment arm.

### Statistical considerations

This was a single-arm phase II study. The primary end point was progression-free survival rate at 6 months (PFR6). A PFR6 of 50% is observed in the unselected patient group when they are treated with taxane monotherapy[12]. To justify further testing of trastuzumab addition to the treatment of patients with HER2-positive CTCs, a PFR6 of 80% in the combination treatment arm was considered sufficient. An A’ Herns single stage phase II design was applied with *p*0 = 50%, *p*1 = 80%, *α* = 0.05 and *β* = 0.20, indicating at least 18 patients with HER2-positive CTCs needed to be included, of which 13 or more patients had to reach PFR6[13]. Secondary endpoints included progression-free survival (PFS) in months, overall survival (OS) in months and percentage of HER2-positive CTCs in relation to outcome. Patients that started with taxane chemotherapy and that had detectable, but no HER2-positive CTCs were used as a control group. Progression-free survival (PFS) and overall survival (OS) were defined as the time from start of taxane treatment to, respectively, the time of clinical or radiological progression of disease or death. Patients were censored at the last date of response evaluation (PFS) or contact (OS). Statistical analyses were performed using IBM SPSS Statistics v25 (IBM Corp., Armonk, N.Y., USA) and for all tests, two-sided p-values of < 0.05 were considered statistically significant. Figures were generated with R version 4.2.2. Reporting is in accordance with the REMARK guidelines [14].

## Results

### Patient characteristics

**Fig. 1:**
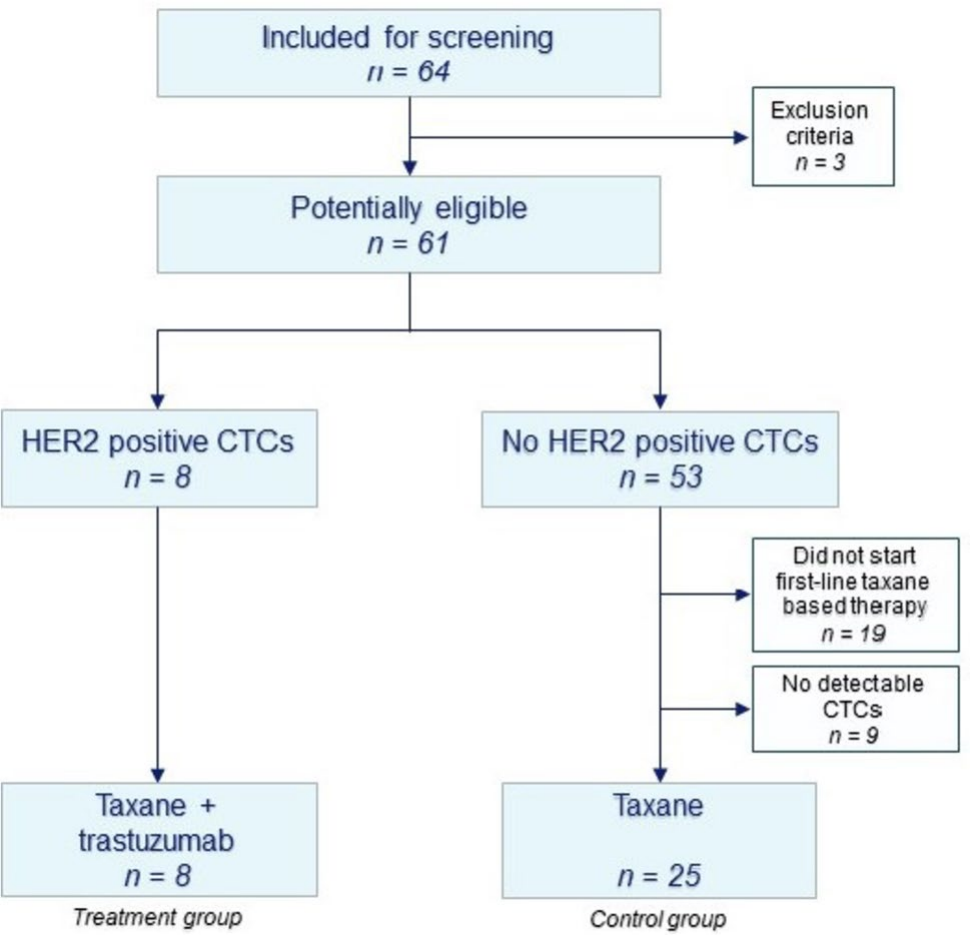
Flowchart of patient inclusion and comparison

In total, 64 patients with a HER2-negative primary tumor were screened for the presence of HER2-positive CTCs, of which 8 patients (7 ER + , 1 triple-negative breast cancer or TNBC) matched this criterion and proceeded to the treatment phase, where they received additional trastuzumab (flowchart Fig. 1). A total of 25 patients had measurable CTCs, but no HER2-positive CTCs and started with taxane therapy, this formed the control group. Baseline characteristics are shown in Table 1**.** In the treatment group, 71% received one or more lines of endocrine therapy before inclusion, whereas the number of previous treatment lines was slightly higher in the control group (Fisher exact test *p* = 0.03), but numerical differences were small (median 0 and 1, Mann–Whitney u test *p* = 0.34). The number of metastatic sites ranged from 1–4 in both groups, with no differences in prevalence of bone, liver and lung lesions. Excluding the patients that had no measurable CTCs, there was a borderline significant difference between median CTC count in the patients that did and that did not have HER2-positive CTCs (*p* = 0.05, Mann–Whitney U test). Of the eight patients with HER2-positive CTCs in the current trial, the number of total CTCs ranged between 1 and 131, of which 1 or 2 were HER2-positive. The immunofluorescence strength on CTCs did not seem to relate to the immunohistochemistry results of either the primary tumor or the metastasis, data per patient is specified in supplementary Table 1.

**Table 1:** Baseline characteristics of patients in the treatment group and in the control group

		Taxane + trastuzumab (*n* = 8)	Taxane only (*n* = 25)	***p***
Age (median, range)		63 (48–75)	59 (41–78)	0.78
ECOG at baseline	0	1	6	
	1	6	11	
	2	1	1	
	Unknown	0	7	0.50
HR subtype	HR-positive	7	24	
	Triple-negative	1	1	0.16
Metastatic site	Bone	6	21	0.57
	Liver	4	10	0.61
	Lung	4	5	0.09
Lines of prior endocrine therapy for metastatic disease (excluding TNBC)	0	2	14	
	1	3	1	
	2	0	4	
	3	2	2	
	4	0	3	0.03
CTC (median, range)		15 (1–131)	5 (1–1047)	0.05
Best objective response	PD	1	6	
	SD	2	10	
	PR	4	5	
	Non evaluable	1	2	
	Unknown	0	2	0.34

### Clinical outcome

**Fig. 2:**
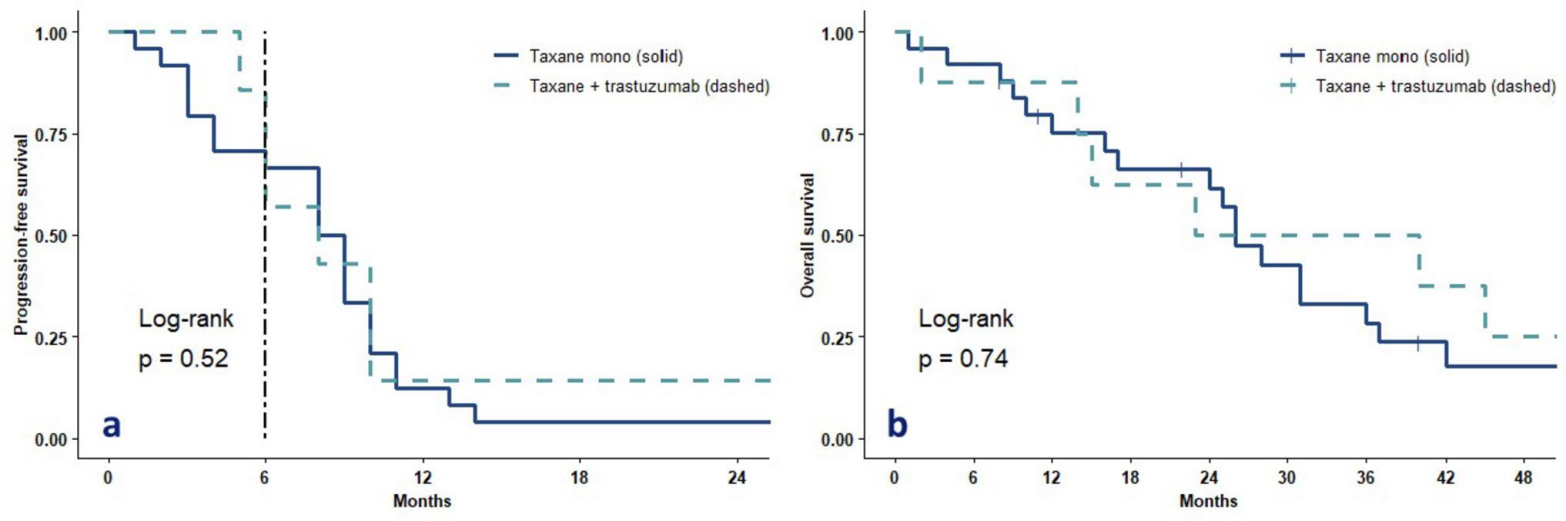
Progression-free survival curves (**a**) and overall survival curves (**b**) of the treatment (taxane + trastuzumab) and the control (taxane mono) group. Dashed line in Fig. 2a represents the 6-months progression-free rate, which was the primary endpoint of this study

The PFR6 was 50% in treatment group (4 out of 8 patients) and 54% in the control group (14 out of 25 patients) (Fig. 2a). Four patients (50%) reached partial response (PR) as best overall response, whereas this was observed in five (20%) of the patients receiving taxane only, but this did not differ significantly (Fishers exact p = 0.34). The median progression-free survival was 8.0 months (95% CI: 2.9 – 13.1 months) for the treatment group and 9.0 months (95% CI: 7.3 – 10.7 months) for the control group and did not differ significantly between the groups (*p* = 0.52, log-rank test). Furthermore, there was no difference in OS (23 months versus 26 months, log-rank test 0.74, Fig. 2b). When dichotomizing all patients with the validated cut-off of 5 CTCs, there was a trend toward worse PFS and OS in patients with more than 5 CTCs (*p* = 0.07 and *p* = 0.16, respectively, log-rank test, supplementary Fig. 1). Of the eight patients receiving additional trastuzumab, six patients received all six cycles of docetaxel. The other patients did not reach six cycles: one patient died after four cycles of treatment from an unknown cause before the first response evaluation and one patient stopped after five cycles of taxane treatment because of toxicity. There were no ≥ grade 4 events reported in this patients. There was no significant decrease of cardiac LVEF in patients treated with trastuzumab (paired samples T-test *p* = 0.50) and all patients had an ejection fraction of > 45% at follow-up.

### Association between CTC-count and presence of HER2-positive CTCs

**Fig. 3:**
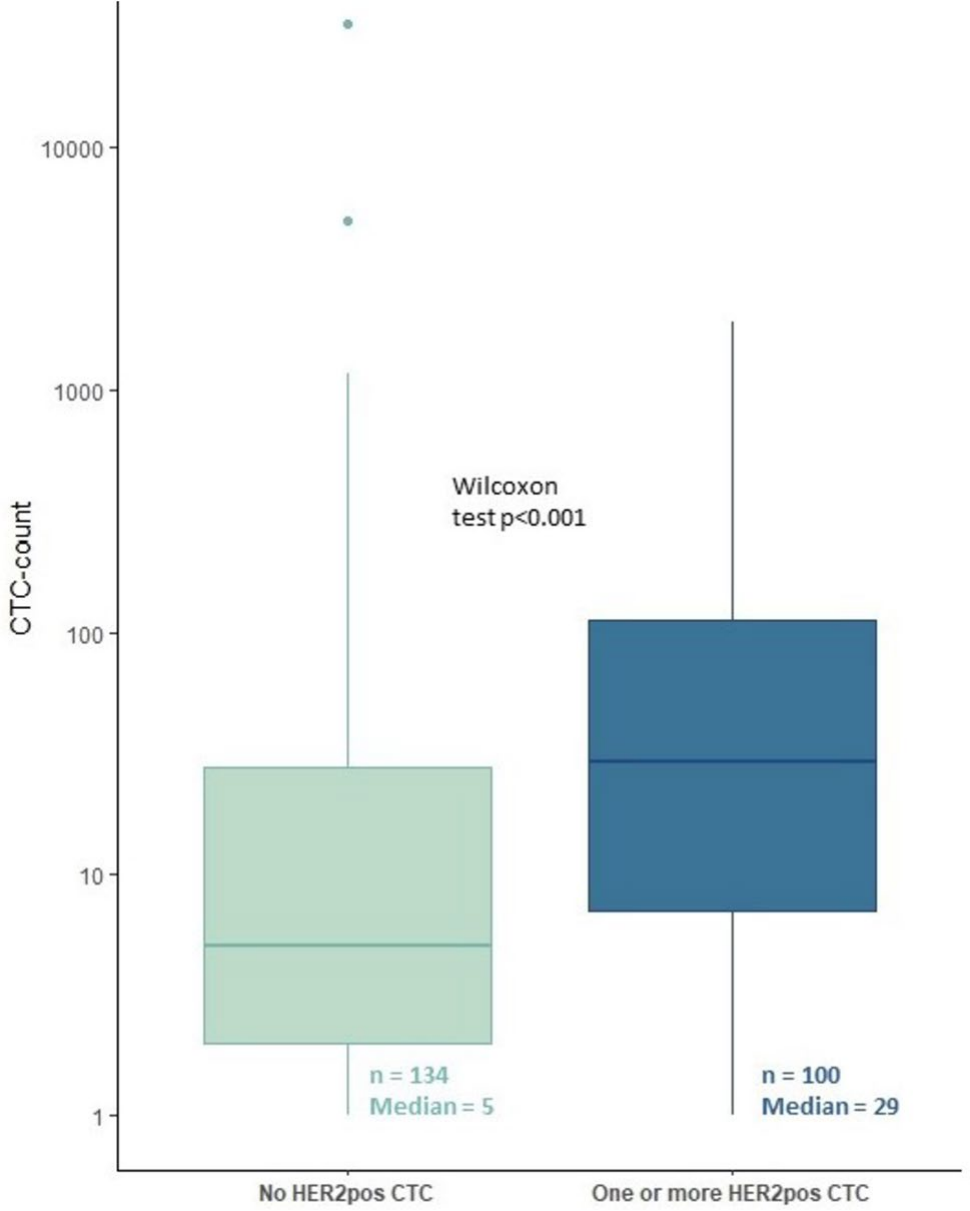
Boxplots showing a significant difference between median CTC-count in the patients with and without detectable HER2-positive CTCs

Because the trial was terminated early, leading to low patient numbers, we wanted to validate the observation of the association between CTC-count and the detection of one more HER2-positive CTC. We therefore repeated this analysis for all samples in HER2-negative MBC patients in the previously reported 06–248 and 09–405 studies, and in the currently running Caremore-AI study (NTR 5121)[15–17]. From these studies, we selected all patients (*n* = 234) that had detectable CTCs before the start of first line endocrine therapy or chemotherapy. Of these, 100 patients (43%) had one or more HER2-positive CTCs, according to the semi-quantative cut-off of 2 + and higher, as described in the methods section. We confirmed a significant higher median CTC-count in samples that had at least one HER2-positive CTC (29 versus 5, *p* < 0.001 Wilcoxon test, Fig. 3). The number of HER2-positive CTCs ranged from 1–117, with a percentage from total number of CTCs ranging from 0.1–100%.

## Discussion

This study aimed to evaluate the efficacy of adding trastuzumab to first-line taxane-based chemotherapy in patients with a HER2-negative primary tumor and HER2-positive circulating tumor cells. Eight patients received trastuzumab, of which four patients achieved the pre-specified primary outcome of PFR at 6 months. Although a numerical increase in partial responses was observed with trastuzumab therapy, the difference was not statistically significant in terms of PFR6, PFS, or overall survival. Despite originally requiring 18 patients to achieve sufficient statistical power, the results from the first eight patients suggest that it would have been highly unlikely to meet the primary endpoint by continuing the study. This would have meant that of the remaining 10 patients included into the study, nine had to show no progression at 6 months. Therefore, the study was terminated early. Even though the study was underpowered as a consequence, we believe that adding trastuzumab to taxane therapy in patients with HER2-positive CTCs is not an effective treatment strategy.

Our study was part of a series of trials evaluating HER2-targeted treatments in HER2-negative patients with HER2-positive CTCs. In Table 2 [18–22], we provide an overview of published and ongoing interventional studies, which employed various methods for CTC enrichment and HER2-status assessment (e.g., immunofluorescence and/or FISH) and different thresholds for HER2-positivity. Across these trials, the median number of patients required for screening to find one patient with HER2-positive CTCs was approximately 9 (ranging from 5 to 20), similar to our trial. While being single-arm phase II trials and utilizing different anti-HER2 targeting agents, overall clinical benefit was lacking in these published trials. Currently, results from the first randomized phase III DETECT-trial (NCT01619111) are awaited, which evaluates addition of the HER2-directed tyrosine kinase inhibitor lapatinib to standard of care in patients with a HER2-negative tumor and HER2-positive CTCs at the start of a new line of treatment. Preliminary results analyzing 105 randomized patients did not show a difference in progression-free survival [18]. Several solutions have been proposed to increase the chance of clinical benefit for these patients.

**Table 2:** Interventional studies performed using HER2 detection in CTCs

Author	Study type	Patient characteristics	Intervention	Primary outcome measure	CTC enrichment method	HER2 detection method	Cut-off for HER2 positivity	Screened (*n*)	HER2 + CTC (n)	Treated (n)	Response
Pestrin et al. 2012	Single-arm phase II	HER2-negative MBC, pre-treated with anthracycline and/or taxane, at least one line of treatment for MBC	Lapatinib	Objective response rate	CellSearch	Immunofluorescence and/or FISH	≥ 50% CTCs 2 + or 3 + OR minimal 1 FISH amplified	139	7	7	No objective responses, median time to progression was 2.4 months
Hainsworth et al. 2016	Single-arm phase II	HER2-neg MBC, ≥ 1 lines of prior chemotherapy	Trastuzumab and pertuzumab	Response after 2 cycles	Ferrofluids (CellSearch)	PRO ONC assay	Value above cutoff of HBD mean + 2.3 sd	283	24	14	1/14 PR, 1/14 SD (with addition of docetaxel), 12/14 PD
Jacot et al. 2019	Single-arm phase II	HER2-neg MBC, ≥ 2 lines of prior chemotherapy	T-DM1	Objective response rate	CellSearch	FISH	≥ 1 HER2 amplified CTC (> 2.2 HER2/CEP17 or > 6 copies HER2)	154	14	11	1 PR, 4 SD, 6 PD
Parsons et al. 2021	Single-arm phase II	HER2-neg MBC, ≥ 1 lines of prior chemotherapy	Vinorelbine and trastuzumab	Objective response rate	Ferrofluids (CellSearch) + OncoCEE	FISH	≥ 1 HER2 amplified CTC (> 2.0 HER2/CEP17)	311	69	20	1 PR, 3 SD, 16 PD
Fehm et al. 2021 (Abstract)	Randomized phase III	HER2-neg MBC, any ine of therapy	Lapatinib + standard of care	CTC clearance rate	CellSearch	Immunofluorescence	≥ 1 HER2 overexpressing CTC (2 + or 3 +)	Numbers not presented in abstract	105	Randomization 1:1 (numbers not presented in abstract)	No difference in CTC clearance (primary outcome). No difference in PFS (secondary outcome)
*MBC* *metastatic breast cancer, HBD* *healthy blood donor, sd* *standard deviation, FISH* *fluorescent *in situ* hybridization, PR* *partial response, SD* *stable disease, PD* *progressive disease*											

First, the threshold for number of evaluable CTCs might be increased to make a more accurate judgment on tissue HER2-status. The concordance rate of HER2 evaluation on CTCs and in tissue ranges around 70% in metastatic breast cancer[10]. In our observational studies, we showed that the median number of CTCs is higher in the samples with one or multiple HER2-positive CTCs, which was observed by other authors as well[23, 24]. This shows either that a minimal number of CTCs is necessary to find this rare sub-fraction, or, alternatively, that immunofluorescence heterogeneity is always present to some extent, and therefore only observed in patients with enough events. It was previously shown that the diagnostic accuracy of CTCs for predicting the primary HER2-positive status of the tumor could increase to 80%, but this was in case of ≥ 50 evaluable CTCs[25]. With the FDA approved and widely applied CellSearch method which was used in this study, 9% of patients had more than 50 evaluable CTCs. In situ hybridization on enumerated CTCs is possible, but this too requires a large amount of evaluable cells. This is analogous to tissue, as the ASCO/CAP pathology guidelines prescribe complete membrane staining should be observed in at least 10% of tumor cells. Weak to moderate staining (2 +) leads to the recommendation of performing a reflex in situ hybridization to confirm HER2 amplification on DNA level and only the patients with verifiable amplification are HER2-positive. The former implies that a minimal number of tumor cells is needed to make a judgment on HER2-status of the tumor[1]. Increasing the threshold of enumerated CTCs for HER2 evaluation would however drastically increase the numbers of patients needed to screen and decrease the number of patients eligible for further treatment.

Second, a more stringent cut-off for HER2-positivity might help in selecting the right patients for anti HER2-targeted therapy. A variable cut-off for HER2-positivity, for example by taking into account the percentage of HER2-positive CTCs or only taking CTCs that score 3 + on immunofluorescence, is proposed as a possible strategy to increase the chance on clinical benefit[26, 27], but increasing the cut-off for HER2-positivity will too increase the numbers needed to screen.

Lastly, since CTC-count in itself is an independent prognostic factor, this can bias reports about the diagnostic accuracy and the prognostic value of the presence of HER2-positive CTCs in HER2-negative primary breast cancer patients[8, 10]. In our trial, the prognostic value of CTC count was not significant, but this is most likely due to the fact that the overall patient cohort is small. As we showed that the patients with detectable HER2-positive CTCs had a higher baseline CTC count and thus worse prognosis, performing a single-arm study will not provide conclusive data, because of the baseline difference in prognosis. The first results from the interventional DETECT-trial confirmed worse overall survival in the patients with detectable HER2 + CTCs in univariable analysis, although this was not significant when corrected for other factors. The most valuable information from treatment effect of adding targeted therapy in these patients would come from stratifying patients according to CTC count and randomize between targeted treatment and control, as in the DETECT-trial. However, the DETECT-trial study ran for ten years across nine including centers in its current form using the same threshold and cut-off as we did, indicating the complexity of completing such a trial, let alone to perform this with a higher threshold or more stringent cut-off. Even if all above barriers would be overcome and a clinical trial would show clinical benefit, than the results would be applicable for a small subset of patients, thereby limiting clinical utility.

Our trial was performed in HER2-negative patients, but mostly in patients with ER-positive disease. The majority of patients with HER2-positive CTCs were therefore treated previously with endocrine therapy. Jordan et al.[28] showed that in this specific ER + population, cultured CTCs showed strong HER2-expression, but further characterization showed that these cells were not ‘addicted’ to the HER2 downstream pathway, like in HER2-amplified tumors. The HER2 overexpressing CTCs were able to interconvert between a HER2-positive and HER2-negative state. This may in part explain the phenomenon of strong HER2 expression observed with immunofluorescence, but limited results of targeting this with anti-HER2 therapy across trials.

With the upcoming new generation of antibody drug conjugates, the paradigm of HER2 evaluation is shifting. Next to treatment in the HER2-positive group, trastuzumab-deruxtecan has recently been also been approved in the so-called ‘HER2low’ subgroup, which is comprised of patients with tumors scoring 1 + or 2 + without amplification by pathology review[29]. The definition of this subgroup has sparked considerable discussion due to the high degree of interrater variability in scoring HER2low on tumor tissue[30]. The potential contribution of CTC evaluation to HER2 assessment remains a topic of future research. One possible approach might be to identify those patients that have no measurable HER2 expression on CTCs and are not expected to respond to trastuzumab-deruxtecan[31]. Furthermore, various methods for single-cell analyses and more linear HER2 assays have been proposed to enhance the characterization of the HER2-low entity[32]. A possible strategy to gain fundamental insights into the presence and evolution of HER2 expression on single CTCs in vivo, is to increase the yield through diagnostic leukapheresis. With this method, multiple liters of peripheral blood are filtered through continuous centrifugation, which yields around 200-fold enrichment of CTCs in metastatic breast cancer patients. This allows for the evaluation of a large number of CTCs and subsequent in-depth investigation on single cells[33].

## Conclusion

Taken together, characterizing HER2 expression on CTCs is possible, but due to their rarity, interpreting overexpression or amplification becomes challenging, especially with low numbers of CTCs. Moreover, it is difficult to distinguish between the prognostic value of CTC count and the predictive value of HER2-positivity in CTCs, thus the interpretation of both observational and interventional trial results is limited. Applying more stringent thresholds and a randomized trial design seem necessary, but this limits trial feasibility and subsequent clinical application. Based on the above considerations, we argue that pursuing the strategy of additional treatment with HER2-targeted agents known to benefit only HER2-positive patients should not be recommended for patients with HER2-positive CTCs. However, the emergence of new and promising antibody–drug conjugates showing clinical benefit in the HER2low subgroup, may open up new potential for clinical utility of CTC evaluation. Nonetheless, analytical and clinical validity of assays first need to be re-evaluated.

### Supplementary Information

Below is the link to the electronic supplementary material.

Supplementary material 1 (DOC 127.5 kb)

## Data Availability

The data generated during the current study are available from the corresponding author upon reasonable request.

## Abbreviations

ASCO/CAP: American Society of Clinical Oncology and the College of American Pathologists
CTC: Circulating tumor cell
ECOG: Eastern Cooperative Oncology Group
FDA: Food and Drug Authority
FISH: Fluorescence In Situ Hybridization
HER2: Human epidermal growth factor receptor 2
LVEF: Left Ventricular Ejection Fraction
RECIST: Response Evaluation Criteria in Solid Tumors
