# Supplementary material for: Trastuzumab and first-line taxane chemotherapy in metastatic breast cancer patients with a HER2-negative tumor and HER2-positive circulating tumor cells: a phase II trial
Source: Breast Cancer Res Treat. 2024 Jan 31;205(1):87–95. doi: 10.1007/s10549-023-07231-4 (PMC11062986; doi:10.1007/s10549-023-07231-4)
Supplement: Supplementary file 1 — Supplementary material 1 (DOC 127.5 kb) [file 10549_2023_7231_MOESM1_ESM.doc]

**Supplementary material**

**Trastuzumab and first-line taxane chemotherapy in metastatic breast cancer patients with a HER2-negative tumor and HER2-positive circulating tumor cells: a phase II trial**

*Journal: Breast Cancer Research and Treatment*

Noortje Verschoor^1^, Manouk K. Bos^1^, Ingeborg E. de Kruijff^1^, Mai N. Van^1^, Jaco Kraan^1^, Jan C. Drooger^2^, Johanna M. Zuetenhorst^4^, Saskia M. Wilting^1^, Stefan Sleijfer^1^, Agnes Jager^1^, John W.M. Martens^1^

^1^Department of Medical Oncology, Erasmus MC Cancer Institute, Rotterdam, The Netherlands

^2^Department of Internal Medicine, Breast Cancer Center South Holland South, Ikazia Hospital, Rotterdam, The Netherlands

^4^Department of Medical Oncology, Franciscus Gasthuis & Vlietland, Rotterdam/Schiedam, the Netherlands

**Correspondence:** [n.verschoor@erasmusmc.nl](mailto:n.verschoor@erasmusmc.nl), ORCID-ID 0000-0003-4465-253X


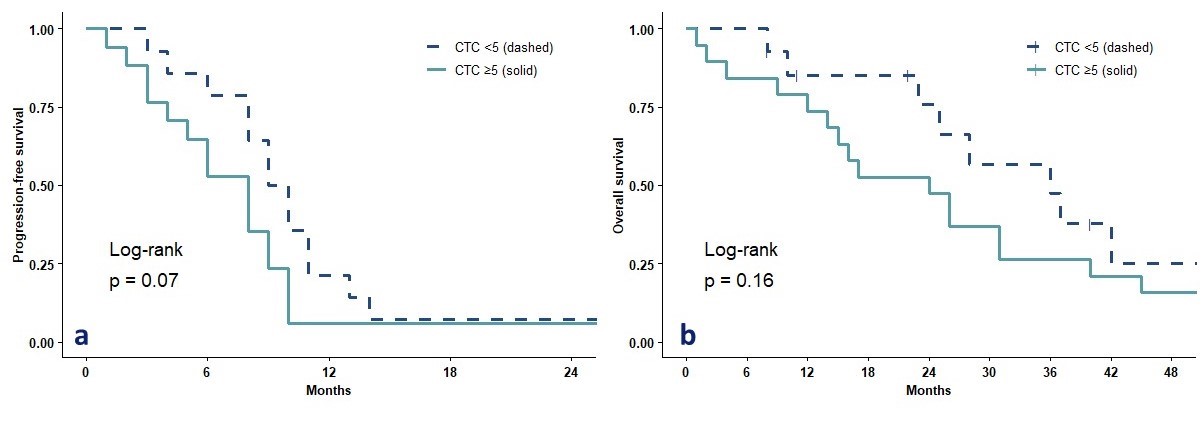


**Supplementary Figure 1:** Progression-free survival curves (2a) and overall survival curves (2b) by CTC-count, dichotomized by the validated cut-off of 5 CTCs.

| **Supplementary table 1: Details on CTC immunofluorescence strength and tumor IHC strength** | | | | | | | |
| --- | --- | --- | --- | --- | --- | --- | --- |
|  | | | | | | | |
| **Subject number** | **Subtype** | **No. of CTC** | **No of HER2+ CTC** | **Strength IF** | **PFS** | **IHC primary** | **IHC metastasis** |
| 1 | HR+ | 11 | 1 | 3+ | 10 | 2+ | ND |
| 3 | HR+ | 120 | 2 | 2+ | 10 | 1+ | ND |
| 10 | HR+ | 1 | 1 | 3+ | 58 | neg | ND |
| 32 | HR+ | 18 | 2 | 2+ | 8 | ND | ND |
| 37 | TNBC | 1 | 1 | 3+ | 6 | 0 | 0 |
| 39 | HR+ | 110 | 1 | 2+ | 2 | ND | 1+ |
| 55 | HR+ | 131 | 1 | 2+ | 6 | 1+ | ND |
| 63 | HR+ | 35 | 2 | 2+ | 5 | 2+ | ND |
| 8 | HR+ | 14 | 0 |  | 3 | ND | 1+ |
| 14 | HR+ | 1 | 0 |  | 11 | 0 | ND |
| 15 | HR+ | 58 | 0 |  | 43 | 0 | 1+ |
| 18 | HR+ | 3 | 0 |  | 4 | 1+ | ND |
| 25 | HR+ | 9 | 0 |  | 9 | 1+ | 1+ |
| 29 | HR+ | 5 | 0 |  | 1 | 0 | 1+ |
| 30 | HR+ | 8 | 0 |  | 4 | 1+ | 1+ |
| 31 | HR+ | 3 | 0 |  | 10 | 1+ | ND |
| 34 | HR+ | 14 | 0 |  | 8 | ND | neg |
| 36 | HR+ | 2 | 0 |  | 10 | neg | ND |
| 38 | TNBC | 10 | 0 |  | 1 | 0 | ND |
| 42 | HR+ | 2 | 0 |  | 9 | 1+ | ND |
| 44 | HR+ | 4 | 0 |  | 13 | 0 | ND |
| 45 | HR+ | 30 | 0 |  | 6 | 0 | 1+ |
| 46 | HR+ | 3 | 0 |  | 3 | neg | neg |
| 47 | HR+ | 1047 | 0 |  | 8 | neg | ND |
| 50 | HR+ | 2 | 0 |  | 8 | 0 | ND |
| 51 | HR+ | 5 | 0 |  | 9 | 0 | 1+ |
| 52 | HR+ | 4 | 0 |  | 9 | neg | ND |
| 53 | HR+ | 6 | 0 |  | 2 | 1+ | ND |
| 54 | HR+ | 1 | 0 |  | 14 | 0 | 2+ |
| 57 | HR+ | 6 | 0 |  | 10 | 0 | 0 |
| 58 | HR+ | 7 | 0 |  | 3 | 0 | ND |
| 61 | HR+ | 1 | 0 |  | 11 | 0 | ND |
| 64 | HR+ | 2 | 0 |  | 8 | 0 | 1+ |
| *Strength IF = semi-quantitative strength of immunofluorescence on CTCs, PFS = progression-free survival in months, IHC = immunohistochemistry, ND = not determined, neg = HER2 not quantified* | | | | | | | |
|
